# *Bacillus cereus*: An Ally Against Drought in Popcorn Cultivation

**DOI:** 10.3390/microorganisms12112351

**Published:** 2024-11-18

**Authors:** Uéliton Alves de Oliveira, Antônio Teixeira do Amaral Junior, Samuel Henrique Kamphorst, Valter Jário de Lima, Fábio Lopes Olivares, Shahid Khan, Monique de Souza Santos, Jardel da Silva Figueiredo, Samuel Pereira da Silva, Flávia Nicácio Viana, Talles de Oliveira Santos, Gabriella Rodrigues Gonçalves, Eliemar Campostrini, Alexandre Pio Viana, Freddy Mora-Poblete

**Affiliations:** 1 Plant Breeding Laboratory, Center for Agricultural Science and Technologies (CCTA), State University of Norte Fluminense Darcy Ribeiro–UENF, Campos dos Goytacazes 28013-602, RJ, Brazil; uelitonalves2011@hotmail.com (U.A.d.O.); amaraljr@uenf.br (A.T.d.A.J.); valter_jario@hotmail.com (V.J.d.L.); moniquesantos47@gmail.com (M.d.S.S.); jardel.figueiredo7@gmail.com (J.d.S.F.); samuelvip26@gmail.com (S.P.d.S.); flaalegre@hotmail.com (F.N.V.); tallesdeoliveira@live.com (T.d.O.S.); rdgabriella@gmail.com (G.R.G.); campostrini@uenf.br (E.C.); pirapora@uenf.br (A.P.V.); 2 Laboratório de Biologia Celular e Tecidual, Universidade Estadual do Norte Fluminense Darcy Ribeiro, Av. Alberto Lamego, 2000, Campos dos Goytacazes 28013-602, RJ, Brazil; fabioliv@uenf.br; 3 Faculty of Agriculture Sciences, Universidade Federal da Grande Dourados (UFGD), Dourados 79800-000, MS, Brazil; shahidkhan@ufgd.edu.br; 4 Institute of Biological Sciences, University of Talca, Talca 34655-48, Chile; morapoblete@gmail.com

**Keywords:** rhizobacteria, bioinoculant, plant growth-promoting bacteria (PGPR), water deficit, *Zea mays* Everta

## Abstract

Despite the development of adapted popcorn cultivars such as UENF WS01, strategies such as bacterial inoculation are being explored to enhance plant resilience to abiotic stress. This study investigates the impact of drought stress on popcorn cultivation. Specifically, the aim was to identify the benefits of *Bacillus cereus* interaction with the drought-tolerant hybrid UENF WS01 for its morphophysiology and growth by comparing inoculated and non-inoculated plants under water-stressed (WS) and well-watered (WW) conditions. This evaluation was conducted using a randomized complete block design in a factorial arrangement. For WS with inoculation samples, there were significant increases in relative chlorophyll content, maximum fluorescence intensity, and agronomic water use efficiency. Chlorophyll content increased by an average of 50.39% for WS samples, compared to a modest increase of 2.40% for WW samples. Both leaf and stem biomass also significantly increased for WS relative to WW conditions. Overall, *B. cereus* inoculation mitigated the impact of water stress, significantly enhancing the expression of physiological and morphological traits, even when paired with a drought-tolerant hybrid.

## 1. Introduction

In the context of climate change, drought is the most pressing threat to food security and human health [1,2]. This abiotic stress induces oxidative, osmotic, and ionic effects that negatively impact crop health [3,4], affecting photosynthetic activity, hormone production, membrane stability, nutrient uptake, and reactive oxygen species (ROS) accumulation. These changes ultimately affect plant growth, development, and productivity [5,6].

Given these challenges, *Zea mays* L. var. Everta is particularly notable for its ability to explode when subjected to high temperatures, a key characteristic of popcorn [7]. Popcorn is widely recognized not only for its sensory properties but also for its favorable nutritional profile, including fiber, vitamins, and minerals, making it a highly attractive product for consumers and solidifying its popularity in the snack market [8,9].

For popcorn, research has highlighted the significant impacts of water stress on various agronomic traits, including early reductions in leaf greenness and root angle and volume, as well as decreases in grain yield, popping expansion, and grain weight [10,11]. By affecting the greenness index, water stress also compromises photosynthetic status and stomatal conductance [12].

Following extensive research on various popcorn genotypes under water stress conditions [10,11,13,14,15], Lima et al. (2021) [8] successfully registered the only drought-tolerant popcorn cultivar in Brazil, named UENF WS01, with the Brazilian Ministry of Agriculture, Livestock, and Food Supply (MAPA).

Developing cultivars adapted to water scarcity is essential; however, management strategies that enhance crop tolerance to abiotic stresses can also improve yields under adverse conditions [16,17]. Among these strategies, applying biostimulants, such as plant growth-promoting rhizobacteria (PGPR), has proven to be a beneficial approach to increasing the productivity of various crops under abiotic stress [18,19,20,21,22].

Within the group of endophytes identified as an option for agricultural purposes, the genus *Bacillus* has emerged as a key player, widely distributed across various plant species, and recognized for its growth-promoting and biocontrol capabilities [23]. In particular, the microorganism *Bacillus cereus*, a cylindrical, Gram-positive bacterium, adapts well to diverse environments [24]. Mobile and capable of forming spores in oxygen, *B. cereus* is found in soil, dust, water, decaying matter, food, and plant roots [25]. Research has demonstrated that *B. cereus* strains promote plant growth in optimal conditions and underwater, salt, and heavy metal stresses [18,26].

Nonpathogenic strains of *B. cereus* offer several promising applications in agriculture [27,28,29,30]. Recent studies have highlighted their effectiveness in controlling phytopathogenic diseases, the bioremediation of contaminated soils, and enhancing tolerance to abiotic stresses [3,31,32,33,34].

Despite the recognized potential of *Bacillus cereus* to stimulate plant growth, research on its interactions with specific plant species remains limited. Specifically, for popcorn (*Zea mays* L. Everta) crops, there has been no investigation into the potential influence of this microorganism in mitigating the adverse effects of water deficit. Therefore, this study aims to explore the beneficial effects on the morphophysiology of the drought-tolerant popcorn hybrid UENF WS01 resulting from its interaction with *Bacillus cereus*. This analysis will consider both water-stressed conditions and normal irrigation conditions. This investigation is particularly relevant given that drought-tolerant genotypes can suffer significant production losses under prolonged or severe water stress conditions.

## 2. Materials and Methods

### 2.1. Inoculum Preparation

The *Bacillus cereus* strain UENF-LMS71 inoculum was prepared using the bacterial stock from the Laboratory of Cellular and Tissue Biology at the State University of Northern Rio de Janeiro (LBCT/UENF). The bacterial strain was inoculated on Petri dishes containing DYGS solid medium [35] using a platinum bacteriological loop. The plates were then incubated for 72 h at 30 °C.

For the pre-inoculum, a bacterial colony was transferred from the Petri dish to a test tube containing 5 mL of DYGS liquid medium using a bacteriological loop. This culture was incubated at 30 °C with orbital shaking at 180 rpm for 24 h. To prepare the final inoculum, 150 μL of the pre-inoculum was pipetted into an Erlenmeyer flask containing 100 mL of DYGS liquid medium and incubated under the same conditions for another 24 h [36]. Following incubation, the optical density (OD) was measured at 600 nm using a spectrophotometer to achieve a target concentration of 1.8 × 10^9^ colony-forming units (CFUs mL^−1^).

### 2.2. Plant Material

The hybrid UENF WS01, registered under the number 46965 in Brazil with the Ministry of Agriculture, Livestock, and Food Supply (MAPA), was utilized in this study. The seeds for this hybrid were produced in the greenhouse at the Research Support Unit of the State University of Northern Rio de Janeiro (UENF) through the crossbreeding of parental lines L76 and L61.

### 2.3. Seed Preparation

The seeds of the popcorn hybrid were disinfested by immersing them in a 3% sodium hypochlorite (NaOCl) solution for 3 min, followed by a 2 min immersion in a 70% ethanol solution. Subsequently, the seeds were thoroughly rinsed in three successive washes with autoclaved distilled water. Pre-germination involved soaking the seeds in distilled water for 5 h. Afterward, the seeds were moistened with distilled water on germination paper and incubated in a BOD (biochemical oxygen demand) incubator at 25 °C for 24 h.

### 2.4. Experiment Implementation

The experiment was conducted in a greenhouse under semi-controlled conditions (rain shelter) at the Research Support Unit of UENF, located in Campos dos Goytacazes, RJ, Brazil.

The experimental design was a factorial arrangement in randomized complete blocks comprising four treatments with nine replicates. The treatments were combinations of the presence and absence of water stress and bacterial inoculation: WSI (inoculated plants under water stress); WSC (control plants under water stress not inoculated); WWI (inoculated plants under normal irrigation); and WWC (control plants under normal irrigation not inoculated).

Seeds subjected to pre-germination were sown in PVC tubes (10 cm in diameter and 150 cm in length) filled with a mixture of 70% Basaplant^®^ substrate and 30% perlite. The inoculation was performed at planting by applying 1 mL of the inoculum directly to the seeds. Non-inoculated treatments received 1 mL of a blank medium to ensure that the only difference between the treatments was the presence or absence of the inoculated bacteria.

Irrigation was halted for the WS treatments 24 days after planting (DAP) and resumed at 40 DAP when the plants reached 40% of field capacity (FC). The plants were maintained at this moisture level for an additional eight days. Conversely, the plants in the WW treatments were maintained at 100% FC throughout the experiment.

To estimate field capacity, the tubes were thoroughly irrigated prior to planting. Subsequently, they were allowed to percolate for 72 h to drain excess water. After this period, the tubes were weighed on a precision scale to determine their maximum water capacity (100%). Simultaneously, substrate samples were oven-dried at 70 °C for 72 h. The field capacity for each tube was calculated by subtracting the weight of the dry substrate (before saturation) from the wet weight after drainage. The tubes were weighed and watered at 2-day intervals throughout the experiment to maintain their respective capacities. The average water content per tube at relative field capacity was 4.772 L (100%) in WW and 3.157 L (40%) in WS samples. Plant weight was not considered when calculating the water capacity of the tubes.

Nutrient provision was facilitated through the complete solution by Hoagland and Arnon (1950) [37], with 224 ppm of nitrogen applied during all irrigation events until the initiation of stress. The nutrient solution provided essential nutrients to the corn under drought conditions, helping to compensate for the limitations in water and nutrient absorption from the soil. This ensured that the plant remained healthy, even under stress, allowing for effective assessments to be conducted. Water potential was monitored using eight Decagon MPS-6 (Decagon, Pullman, WA, USA) tensiometers, with four allocated to each water condition (WC), placed in the tubes at a depth of 20 cm. For the WS condition, irrigation was halted at 24 DAP, reaching 40% of FC at 40 DAP and the permanent wilting point (−1.5 MPa) at 44 DAP (Supplementary Material—Figure S1).

The climatic conditions—temperature (°C), relative humidity (%), and photosynthetically active radiation (kJ/m^2^)—were monitored using a WatchDog^®^ meteorological station. Throughout the experiment, the average daily temperature ranged from 23.76 °C to 32.50 °C and humidity levels varied from 56.48% to 75.00%. Solar radiation had an average intensity that ranged from 423.08 to 185.44 µmol m^−2^ s^−1^ (Supplementary Material—Figure S2).

### 2.5. Physiological Traits

All physiological traits described below were assessed 49 days after planting (DAP), corresponding to the V12 phenological stage. Measurements were conducted on the middle third of the last fully developed leaf between 10:00 h and 14:00 h.

#### 2.5.1. Leaf Pigments and N Balance Index

The relative chlorophyll content (Chl), anthocyanin leaf content (Anth), flavonoid content (Flv), and nitrogen balance index (NBI) were measured using a portable Dualex^®^ meter from Scientific (model FORCE-A, Orsay, France).

#### 2.5.2. Efficiency of PSII (Chlorophyll Fluorescence)

Chlorophyll fluorescence, a measure of energy capture efficiency by photosystem II (Fv/Fm) centers, was evaluated using the MultispeQ v1.0 device (Photosynq Inc., East Lansing, MI, USA). In addition, this variable was also measured with a portable fluorometer (FluorPen FP 100, Photon Systems Instruments–PSI, Drásov, Czech Republic) that recorded transient OJIP fluorescence. This assessment was performed on the adaxial surface of the leaves, following a 20 min dark adaptation period.

#### 2.5.3. Gas Exchange

Gas exchange was assessed using an IRGA gas analyzer (Li-Cor 6400, LiCor, Lincoln, NE, USA), with an external CO_2_ supply of approximately 400 µL L^−1^ and an irradiance of 1500 μmol m^−2^ s^−1^. The following parameters were evaluated: net photosynthetic rate (A, mol CO_2_ m^−2^ s^−1^), stomatal conductance (*gs*, mol H_2_O m^−2^ s^−1^), intercellular CO_2_ concentration (Ci, μmol CO_2_ mol^−1^), and transpiration rate (E, mmol H_2_O m^−2^ s^−1^).

#### 2.5.4. Leaf Water Status and Agronomic Water Use Efficiency

The relative water content (RLWC) was determined using the plant material’s fresh, turgid, and dry weights. Leaves were precisely cut into 1 cm diameter discs using a circular cutter and immediately weighed to obtain the fresh weight (FW) on a precision scale. The leaf discs were then immersed in distilled water bottles for 24 h. After this period, the surfaces of the leaf discs were dried on paper towels and reweighed to determine the saturated weight (SW).

The samples were subsequently placed in a forced-air oven at 70 °C for 72 h to obtain the dry weight (DW). The relative water content (%) was calculated using the formula RLWC = [FW − SW/SW − DW] * 100 (Turner, 1981). The specific leaf area (SLA) was determined using the same leaf discs, calculated by the formula $\mathrm{SLA}=\frac{A}{\mathrm{DW}}$, expressed in cm^2^ g^−1^.

The total amount of water transpired by each plant, or cumulative plant evapotranspiration (ET, dm^3^ plant^−1^), was monitored throughout the growth cycle. Before each irrigation, the tubes were weighed, and the upper surface of each tube was covered with a plastic film (punctured with small holes to allow gas exchange) to prevent direct evaporation from the substrate surface. The agronomic water use efficiency (WUE_agro_, g kg^−1^) was calculated using the formula ${\mathrm{WUE}}_{\mathrm{agro}}=\frac{\mathrm{BA}}{\mathrm{ET}}$. Using data obtained with the IRGA gas analyzer, intrinsic water use efficiency (WUE_int_ = A/gs-μmol CO_2_ mol^−1^ H_2_O) and instantaneous water use efficiency (WUE_inst_ = A/E-μmol CO_2_ mmol^−1^ H_2_O) were also calculated.

### 2.6. Morphological Traits

The traits listed below were measured on all plants, and the averages for each treatment were subsequently calculated.

Plant height (PH, cm), leaf length (LL, cm), and leaf width (LW, cm) were measured using a tape measure. Stem diameter (SD, mm) was assessed using a digital caliper.

After evaluating these traits, the plants were separated into leaves and stems, placed in paper envelopes and stored in an oven at 70 °C until their weight stabilized. The average dry biomass for the leaves (LB, g) and stems (SB, g) was then determined using a precision analytical balance.

### 2.7. Measurement of Stomatal and Epidermal Cell Density

The adaxial and abaxial epidermal surfaces of the middle third of the last developed leaf specifically delineated between the central vein and the tip, were coated with nail polish. After allowing the polish to dry for 10 min, the dried enamel layer was carefully removed using adhesive tape and then transferred to a glass slide. The number of stomata (*s*) and epidermal cells (*ec*) was counted under a microscope equipped with a 40x objective lens. Each leaf surface (adaxial and abaxial) was analyzed in three microscope fields. This methodology follows the protocol outlined by Radoglou and Jarvis (1990) [38].

Adaxial and abaxial stomata density (SD_AD_ and SD_AB_, expressed as stomata mm^−2^) and adaxial and abaxial epidermal cell density (ECD_AD_ and ECD_AB_, expressed as cells mm^−2^) were calculated using the formulae $\mathrm{SD}=\frac{s}{0.63}$ and $\mathrm{ECD}=\frac{ec}{0.63}$, where 0.63 mm^−2^ is the surface area of the microscope (radius of 0.22 mm). The adaxial and abaxial stomatal indices (SI_AD_ and SI_AB_, expressed as percentages) for each leaf surface were estimated using the expression $\mathrm{SI}=100\;\left(\frac{\mathrm{SD}}{\mathrm{EC}}\right)$.

### 2.8. Analysis of Root Traits

The root material was sectioned into five equal sections (RB) along the height of the tubes from the upper to the lower end: 0–30 cm (a), 30–60 cm (b), 60–90 cm (c), 90–120 cm (d), and 120–150 cm (e). These sections were washed with water to remove any adhering substrate, followed by a rinse with distilled water. The roots were superficially dried with paper towels and stored in a refrigerator at 4 °C for later processing.

Each root section was placed in an A4 acrylic tray containing approximately 1.0 L of water (0.5 to 1.0 cm deep), ensuring that the root samples were spread out to minimize overlap for photographing with a digital camera. The images captured were then processed using ImageJ software, version 1.5, and root morphology was analyzed with GiA Roots software, version 4.5 [39].

The specific root lengths (*SRLs*) for each soil section (*SRL_sample_*, m g^−1^) were calculated from the analyzed images using the formula $SRL_{sample}=TRL_{sample}/RB_{sample}$, where *TRL_sample_* represents the total length of the root segments (m) captured in the photographs and *RB_sample_* refers to the dry biomass of these root segments (g) [40]. The total *SRL_sample_* (m g^−1^) of each segment was determined by applying the equation $SRL_{sec}=\frac{SRL_{sample}{*\mathrm{RB}}_{\sec}}{{\mathrm{RB}}_{\mathrm{sample}}}$. The root weight density of each soil section (*RWD_sample_*, g m^−3^) was calculated following the method proposed by Elazab et al. [40], using the formula $RWD_{sec}=RB_{sec}/\pi *R^{2}*L$, where *RB_sec_* is the dry biomass of the root in the soil section (g), R is the tube radius (0.07 m), and L is the length of each tube segment (0.30 m).

### 2.9. Data Analysis

Individual analysis of variance was performed using the model *Y_ij_* = *m* + *BC_i_* + *B_j_* + *e_ij_*, where *Y_ij_* is the observation of genotype *i* in block *j*, *m* is the overall mean, *BC_i_* is the fixed effect attributed to bacterium I, *Bj* is the random effect of block *j*, and *e_ij_* is the experimental error associated with the observation *Y_ij_*, assuming it is normally independently distributed (NID) with a mean of 0 and variance *σ*^2^.

Subsequently, a combined analysis of variance was conducted to determine potential interactions between the genotypes with the two water availability conditions, following the model *Y_ijk_* = *m* + *B/WC_jk_* + *WC_j_* + *BC_i_* + BC_i_*WC_ij_ + *e_ijk_*, where *Y_ijk_* is the observation of genotype *i* in water condition *j* in block *k*, *m* is the overall mean, *B/WC_jk_* is the effect of block *k* in water condition *j*, *WC_j_* is the fixed effect of water condition *j*, *BC_i_* is the fixed effect of bacterium *I*, BC*WC_ij_ is the fixed effect of the interaction between bacterium i and water condition *j*, and *e_ijk_* is the experimental error associated with the observation *Y_ijk_*, with NID (0, *σ*^2^).

The proportional reduction (%) of each trait, given the comparison between the treatments within each water condition, was calculated using the expression $100-\left[\left(\frac{YWSI\;or\;YWWI}{YWSC\;or\;YWWC}\right)*100\right]$, where *Y* is the overall mean of the trait in the treatments. Subsequently, the means of the treatments were compared using Tukey’s test at a 5% probability level. Statistical analyses were conducted using the GENES computer program [41], while graphical representations were generated using the statistical software R (R Core Team, version 4.2.2) [42].

## 3. Results

The traits Chl, Flav, NBI, Fm, Fv/Fo, A, *gs*, and RLWC exhibited highly significant differences due to the effect of inoculation with *Bacillus cereus*. Anth, Fm/Fo, Fv/Fm, Ci, WUEint, and WUEinst showed significance at *p* ≤ 0.05 for this source of variation. However, Fv, E, NPQT, PHI2, and WUEagro did not differ significantly at *p* < 0.05 for this source of variation (Supplementary Material—Table S1).

For the source of variation ‘water condition’ (WC), significant traits at *p* < 0.01 included Chl, Flv, Anth, NBI, Fm, Fv, Fm/Fo, Fv/Fo, A, *gs*, Ci, E, PHI2, and WUEint, while Fv/Fm and NPQT demonstrated significance at *p* < 0.05. On the other hand, RLWC and WUEinst did not differ significantly at *p* < 0.05.

A significant interaction between the bacterium and water condition (BC*WC) was observed for some physiological traits. Analysis of variance for this source of variation revealed significant differences for Chl, Flv, and Fm at *p* < 0.01 and Anth and Fv/Fo at *p* < 0.05. The other traits did not show significant differences at *p* < 0.05 (Supplementary Material—Table S1).

**Figure 1:**
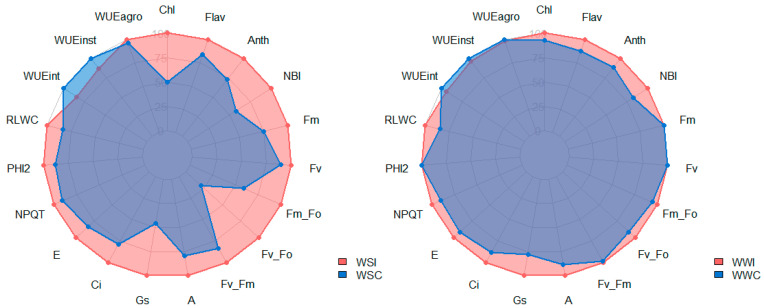
Comparison of the percentage reduction (%) in physiological traits between treatments under water stress (WSI and WSC) and well-watered (WWI and WWC) conditions. Relative chlorophyll content (Chl), leaf anthocyanin content (Anth), flavonoids (Flv), nitrogen balance index (NBI), maximum fluorescence intensity (Fm), variable fluorescence (Fv), basal quantum production of non-photochemical processes in PSII (Fm/Fo), the potential quantum efficiency of PSII (Fv/Fo), potential quantum yield (Fv/Fm), net photosynthetic rate (A), stomatal conductance (Gs), intercellular CO_2_ concentration (Ci), transpiration rate (E), non-photochemical quenching (NPQT), the quantum yield of PSII (PHI2), relative leaf water content (RLWC), intrinsic water use efficiency (WUEint), instantaneous water use efficiency (WUEinst), and agronomic water use efficiency (WUEagro).

Inoculation with *B. cereus* resulted in a percentage increase in all evaluated physiological traits in both WS and WW conditions, except for WUEint and WUEinst in WS samples and Fm, WUEint, WUEinst, and WUEagro in WW samples (Figure 1).

Compared with the control plants under water stress (WSC), the inoculated plants under the same condition (WSI) exhibited significant percentage increases in several physiological traits. Specifically, Chl increased by 50.39%, Anth by 26.63%, NBI by 42.74%, Fm/Fo by 41.11%, Fv/Fo by 78.74%, A by 20.49%, *gs* by 54.55%, and Ci by 21.61%. Under full irrigation conditions, the inoculated plants (WWI) also showed increases compared to the well-watered controls (WWC), though the gains were less pronounced. Notable increases included Flav by 10.91%, Anth by 13.90%, NBI by 17.43%, A by 11.60%, *gs* by 22.22%, Ci by 12.65%, and RLWC by 15.74% (Figure 1).

**Figure 2:**
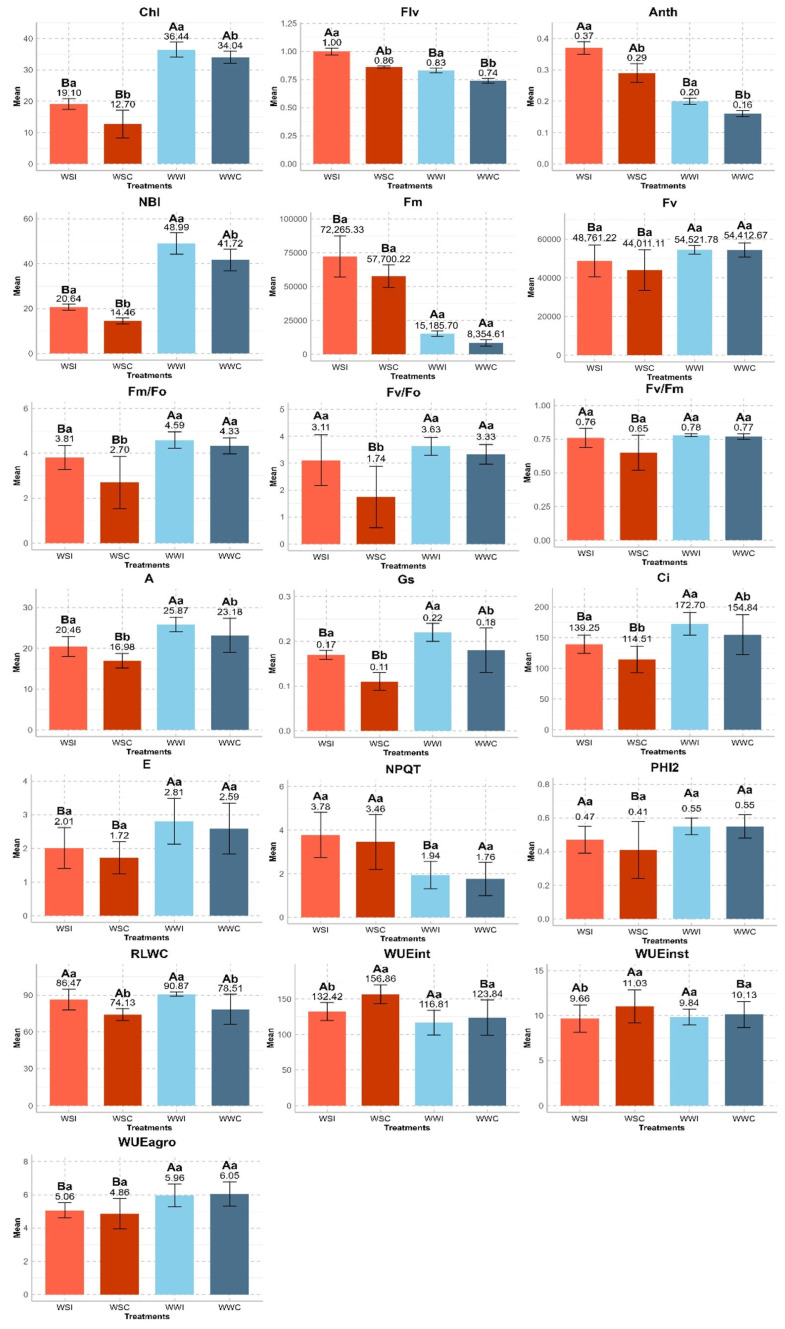
Comparison of means of physiological traits evaluated in hybrid UENF WS01 inoculated with *Bacillus cereus* under two water conditions. Uppercase letters indicate significantly different treatments between water conditions (WSI*WWI and WSC*WWC), and lowercase letters represent significantly different treatments within the water condition (WSI*WSC and WWI*WWC) at the 5% level by Tukey’s test. Error bars show the standard deviation. Relative chlorophyll content (Chl), leaf anthocyanin content (Anth), flavonoids (Flv), nitrogen balance index (NBI), maximum fluorescence intensity (Fm), variable fluorescence (Fv), basal quantum production of non-photochemical processes in PSII (Fm/Fo), the potential quantum efficiency of PSII (Fv/Fo), potential quantum yield (Fv/Fm), net photosynthetic rate (A), stomatal conductance (Gs), intercellular CO_2_ concentration (Ci), transpiration rate (E), non-photochemical quenching (NPQT), the quantum yield of PSII (PHI2), relative leaf water content (RLWC), intrinsic water use efficiency (WUEint), instantaneous water use efficiency (WUEinst), and agronomic water use efficiency (WUEagro).

All physiological traits demonstrated a significant increase with the inoculation of *B. cereus* in plants compared to the respective control groups, except for the variables related to water status: WUEint, WUEinst, and WUEagro (Figure 2).

Regarding actual values, the traits related to leaf pigments, chlorophyll fluorescence, and gas exchange exhibited statistically significant differences within and between the treatments across both WCs. Specifically, Chl, Flav, Anth, NBI, and A all demonstrated increases. In the WS condition, Chl increased by an average of 6.30 in WSI compared to the control, while, for the WWI group, the increase was 2.40. Flavonoids increased by an average of 0.17 in the WSI group and 0.08 in the WWI group. Leaf anthocyanin content increased by an average of 0.07 in the WSI group and 0.02 in the WWI group. The nitrogen balance index saw an average increase of 6.18 in the WSI group and 7.27 in the WWI group compared to their respective controls. The net photosynthetic rate increased by an average of 3.48 mol CO_2_ m^−2^ s^−1^ in the WSI group and 2.69 mol CO_2_ m^−2^ s^−1^ in the WWI group.

Transpiration rate and Fv varied only between water conditions, while Fm/Fo and Ci exhibited significant differences within and between water conditions, specifically under WS. Stomatal conductance and PHI2 differed between the WSC and WWC groups and within the WS condition. Maximum fluorescence intensity and Fm/Fo showed differences between the WSC and WWC groups and within the WS condition. However, Fv/Fm differed only between the WSC and WWC groups. Non-photochemical quenching displayed a significant difference only between the WSI and WWI groups.

In the WSI treatment, E increased by 0.29 mmol H_2_O m^−2^ s^−1^ compared to the control, while, in the WWI treatment, the increase was 0.87 mmol H_2_O m^−2^ s^−1^. Variable fluorescence showed an average increase of 0.02 [a.u.] in the WSI group and 0.12 [a.u.] in the WWI group. The basal quantum production of non-photochemical processes in PSII increased by an average of 1.11 [a.u.] in the WSI group and 1.63 [a.u.] in the WWI group. Intercellular CO_2_ concentration experienced an average increase of 24.74 µmol CO_2_ mol^−1^ in the WSI group and 40.33 µmol CO_2_ mol^−1^ in the WWI group compared to the control. Stomatal density increased by 0.06 mol H_2_O m^−2^ s^−1^ in the WSI and WWI groups. The quantum yield of PSII recorded a mean increase of 0.05 [a.u.] in the WSI group and 0.13 [a.u.] in the WWI group. Maximum fluorescence intensity exhibited a mean increase of 14,565.11 [a.u.] in the WSI group and 12,077.11 [a.u.] in the WWI group compared to the WSC and WWC groups, respectively. The increases for Fv/Fo, Fv/Fm, and NPQT were 1.37 [a.u.], 0.11 [a.u.], and 0.32 [a.u.] in the WSI group and 0.3 [a.u.], 0.01 [a.u.], and 0.18 [a.u.] in the WWI group, respectively.

Regarding water status-related traits, RLWC displayed significant differences only within WCs. Intrinsic water use efficiency differed between treatments in the control group and within the WS condition. Instantaneous water use efficiency showed differences between treatments within WS, while WUEagro differed only between WCs. In the WS condition, RLWC increased by an average of 12.34 compared to the control, while, in the WW condition, the increase was 4.38. Intrinsic water use efficiency displayed an average increase of 15.56 μmol CO_2_ mol^−1^ H_2_O in WS conditions and 33.02 μmol CO_2_ mol^−1^ H_2_O in WW conditions compared to the control. Instantaneous water use efficiency recorded an average increase of 1.17 μmol CO_2_ mmol^−1^ H_2_O in WS conditions and 0.31 μmol CO_2_ mmol^−1^ H_2_O in WW conditions. Agronomic water use efficiency saw an average increase of 0.17 kg m^−3^ in WS conditions and 1.19 kg m^−3^ in WW conditions (Figure 2).

Analysis of variance revealed significant differences for the 19 morphological traits evaluated in hybrid UENF WS01. Traits that exhibited highly significant differences (*p* < 0.01) concerning the source of variation ‘bacterium’ (B) were LW, LB, SB, SLA, SIAB, SIAD, and RWDC, while LL, SDAB, SDAD, and MNRa showed significance at *p* < 0.05. Plant height, SD, ECDAB, ECDAD, MNRb, MNRc, MNRd, and SRLe did not show significant differences at *p* < 0.05 (Supplementary Material—Table S2).

Considering the water conditions as the source of variation, significant traits at *p* < 0.01 included PH, SD, LW, LB, SB, SDAB, ECDAB, SIAB, MNRb, MNRc, and SWDc, while LL, ECDAD, MNRd, and SRLe showed significance at *p* < 0.05. Specific leaf area, SDAD, SIAD, and MNRa did not show significant differences at *p* < 0.05 (Supplementary Material—Table S2).

Regarding the interaction between the bacterium and water condition (BC*WC), analysis of variance showed a significant difference only for RWDc at *p* < 0.05 (Supplementary Material—Table S2).

**Figure 3:**
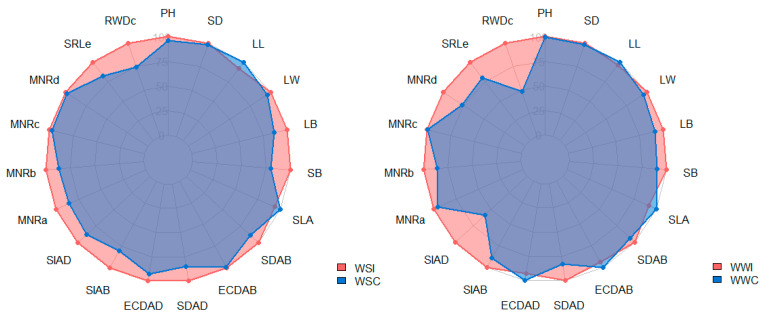
Comparison of the percentage reduction (%) in morphological and root traits between water stress (WSI and WSC) and well-watered (WWI and WWC) conditions. Plant height (PH), stem diameter (SD), leaf length (LL), leaf width (LW), leaf biomass (LB), stem biomass (SB), specific leaf area (SLA), abaxial stomata density (SDAB), abaxial epidermal cell density (ECDAB), adaxial stomata density (SDAD), adaxial epidermal cell density (ECDAD), abaxial stomatal index (SIAB), adaxial stomatal index (SIAD), mean root number (MNR, sections a, b, c, and d), specific root length (SRLe), and root weight density (RWDc).

The presence of inoculation mitigated the impact of water deficit for almost all physiological traits, with LL and SLA as exceptions. Compared with the WSC condition, traits that exhibited the highest percentage increases in the WSI condition were LB (13.33%), SB (20.24%), SDAB (11.45%), SDAD (14.72%), SIAB (19.96%), MNRa (14.43%), SRLe (16.75%), and RWDc (25.18%) (Figure 3).

In the WW condition, the presence of inoculation enhanced PH, SD, LB, SB, SDAB, SDAD, SIAB, and SAAD, with increases of 0.15%, 1.00%, 4.30%, 8.67%, 9.53%, 6.93%, 17.12%, 10.72%, and 41.29%, respectively (Figure 3). All root traits increased with the inoculation with *B. cereus* in both water conditions (Figure 3).

**Figure 4:**
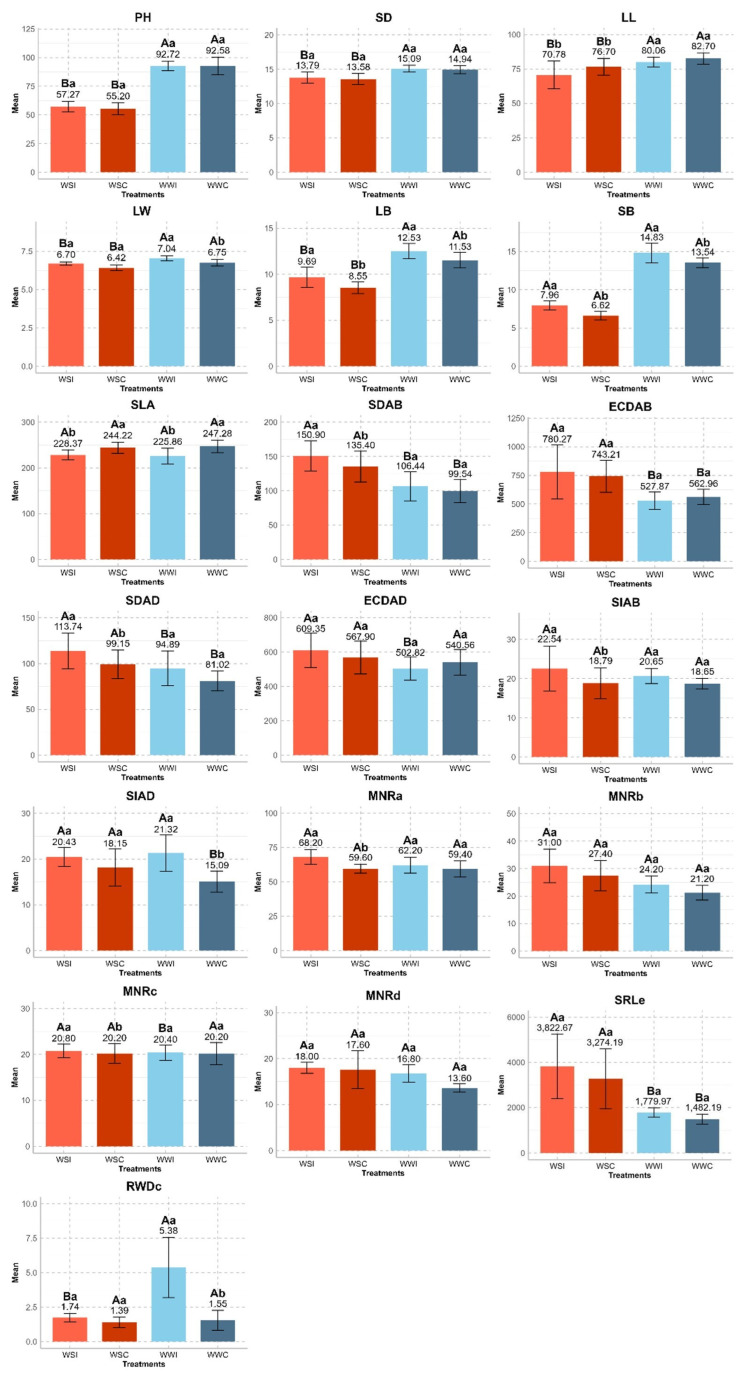
Comparison of means of morphological traits evaluated in hybrid UENF WS01 inoculated with *Bacillus cereus* under two water conditions. Uppercase letters indicate significantly different treatments between water conditions (WSI*WWI and WSC*WWC), and lowercase letters represent significantly different treatments within water conditions (WSI*WSC and WWI*WWC) at a 5% probability level by Tukey’s test. Error bars show the standard deviation. Plant height (PH), stem diameter (SD), leaf length (LL), leaf width (LW), leaf biomass (LB), stem biomass (SB), specific leaf area (SLA), abaxial stomata density (SDAB), abaxial epidermal cell density (ECDAB), adaxial stomata density (SDAD), adaxial epidermal cell density (ECDAD), abaxial stomatal index (SIAB), adaxial stomatal index (SIAD), mean number of roots (MNR, sections a, b, c, and d), specific root length (SRLe), and root weight density (RWDc).

Except for LL, SLA, ECDAB, and ECDAD, all other morphological traits evaluated showed significant increases with inoculation in both water conditions compared to the non-inoculated controls (Figure 4).

Comparing the morphological traits related to plant growth, LB and SB were the only traits that displayed significant differences within and between water conditions. Plant height and SD only showed differences between water conditions, while LW differed both between and within each water condition. Specific leaf area differed only within each water condition.

In the water deficit condition, LB and SB increased by 1.14 g and 1.34 g, respectively, when inoculated with *B. cereus*. In contrast, under well-irrigated conditions with the presence of the bacterium, increases of 1.00 g and 1.29 g were observed compared to the absence of inoculation. Plant height increased by 2.07 cm on average with inoculation in the WS condition and by 0.14 cm in the WW condition. Stem diameter increased by 0.21 mm in the WSI group and 0.15 mm in the WWI group. Leaf width saw an average increase of 0.28 cm in the WSI group and 0.29 cm in the WWI group. Conversely, LL and SLA demonstrated reductions of 5.92 cm and 15.85 cm^2^ g^−1^ in the WSI group and 2.64 cm and 21.42 cm^2^ g^−1^ in the WWI group, respectively.

Regarding epidermal cell and stomatal density, SDAB and SDAD differed between and within the WSI and WSC water conditions. Abaxial epidermal cell density exhibited differences only between water conditions, whereas ECDAD showed variations only in the WSI and WWI groups. Abaxial stomatal index varied only within the WS condition, while SI_AD_ differed only between water conditions for the absence of inoculation and within the WW condition for treatments WWI and WWC.

For the SDAB trait, there was an average increase of 15.50 in the WS condition and 6.90 in the WW condition. Adaxial stomata density increased on average by 14.59 in the WS condition and 13.87 in the WW condition compared to the control. For SIAB and SIAD, there was an average increase of 3.75 and 2.28 in the WS condition and 1.99 and 6.23 in the WW condition, respectively. Abaxial and adaxial epidermal cell density increased by 7.06 and 41.45 in the WS condition and decreased by 35.09 and 37.74 in the WW condition, respectively.

Regarding root traits, MNRa differed significantly only within the water conditions, specifically for the WS condition. The mean number of roots (sections b and d) showed no significant differences between treatments. In the case of section c, the mean root number displayed variations only between water conditions when inoculated and within the WS condition only with inoculation. Specific root length (section e) demonstrated significant differences between water conditions, while RWDc differed only between water conditions with inoculation and within the WW condition, also with inoculation.

For MNRa, an average increase was observed in the WS condition of 8.60 cm in the WW condition of 2.80 cm. Regarding MNRc, there was an average increase of 0.60 cm in the WS and 0.20 cm in the WW conditions. In the WS condition, SRLe showed an average increase of 542.48 compared to the control, while, in the WW condition, the increase was 297.78. As for RWDc, an average increase of 0.35 in the WS and 3.83 in the WW conditions was recorded compared to the control (Figure 4).

## 4. Discussion

Considering only the comparison of water conditions without analyzing the influence of *B. cereus*, the greater accumulation of flavonoids and anthocyanins (Flav and Ant) under water deficit conditions was due to the osmoprotective effect of the plant. This response increased antioxidant capacity under stress conditions, leading to elevated ROS production, which helped preserve the photosynthetic apparatus and cellular components from oxidative damage, thus facilitating drought resilience [43,44].

Furthermore, disregarding the influence of the inoculant, the imposition of a water deficit resulted in a significant reduction in chlorophyll content and the nitrogen balance index (Chl and NBI). This decrease, which was also statistically significant (*p* < 0.05), led to a reduction in the photosynthetic rate, which, in turn, resulted in less growth and production of the plants, as indicated by our results and corroborated by previous studies [9,43].

The WS condition also affected Chlorophyll fluorescence-related traits, resulting in increased Fm and NPQT. In contrast, there were reductions in Fm/Fv, Fv/Fo, Fv/Fm, and PHI2. The increase in NPQT reflects the plant’s capacity for heat dissipation when there is excess light energy [45]. High levels of NPQT can induce a low electron transport rate (ETR), preventing the formation of ROS, which can damage photosystem II (PSII) or inhibit the repair of its reaction centers [46].

All chlorophyll fluorescence traits evaluated in this study showed a greater increase in the WSI group compared to plants in the WSC, WWI, and WWC groups. These results suggest a beneficial effect of *B. cereus* in mitigating damage caused by water stress, leading to the greater adaptation of popcorn to this adverse condition. Similar effects were observed in common corn by Bittencourt et al. (2023) [47], in common corn and sorghum by Aquino et al. (2019) [48], and in soybean, rice, and wheat by Kulkova et al. (2023) [25].

Navarro-Torre et al. (2023) [49] reported that *B. cereus* contributes to greater plant water conservation, improves photosynthetic efficiency, reduces oxidative stress, and preserves cellular integrity. These mechanisms enable plants to maximize light energy capture, decrease ROS production, and maintain cellular structure, thus enhancing survival and productivity in water-restrict environments [50].

Popcorn plants of the UENF WS01 genotype exhibited lower gas exchange rates in the WSC group. The decrease in stomatal conductance, intercellular CO_2_ concentration, and transpiration rate imposes limitations on photosynthesis due to their direct influence on CO_2_ availability. These reductions also lead to stomatal limitations, as wider stomatal openings allow for greater CO_2_ diffusion into the substomatal chamber [51]. Reduced stomatal conductance decreases heat loss via transpiration, resulting in higher leaf temperatures that can further damage the thylakoid membranes, as the photochemical efficiency of PSII is compromised [52].

Inoculation with *B. cereus* significantly increased transpiration, and the enhancement in photosynthesis was often linked to higher chlorophyll content and increased transpiration rates [53]. Similarly, sugarcane plants inoculated with *Bacillus subtilis* exhibited higher gas exchange rates, suggesting an adjustment in stomatal regulation to mitigate the effects of water stress [54]. Plants with improved photosynthetic efficiency often exhibit enhancements such as increased photosynthetic rates, greater biomass production, increased growth and development, and greater resilience to adverse conditions. These advantages contribute to more robust and productive plants that perform better even under environmental stressors such as drought and heavy metal contamination.

Among the impacts of water stress, the observed increase in intrinsic water use efficiency (WUEint) is primarily attributed to a significant reduction in plant transpiration, driven by a decrease in green leaf area due to water scarcity. Our results indicate a notable increase in WUEint under water stress conditions (*p* < 0.01), suggesting that plants optimize their water usage during periods of limited availability. This enhancement in water use efficiency can lead to more effective transpiration, which aids leaf cooling [54]. Taiz and Zeiger (2017) [55] noted that climatic conditions significantly influence gas exchange. Thus, the ability of the plants to maintain gas exchange efficiency, even under stress, is crucial for their resilience and overall performance [56]. The WS condition adversely affected plant morphological traits, including PH, SD, LL, LW, LB, SB, and SLA. A reduction in leaf area under water stress is often associated with increased production of abscisic acid (ABA) in plants, which can stimulate ROS generation, thereby damaging cells and curtailing plant growth and leaf expansion [57,58,59]. In this context, Kumdee et al. (2023) [60], evaluating morphological and physiological attributes of common corn plants under water deprivation, demonstrated that reducing light capture through diminished leaf area decreases cell division and dry matter accumulation.

Studies by different authors have highlighted the ability of *Bacillus cereus* strains to effectively stimulate plant growth under both usual and adverse environmental conditions [18,32,61,62]. Plant growth promoters can enhance growth under drought stress by producing salicylic acid, improving hormonal balance, and increasing phosphorus uptake [63]. Bandeppa et al. (2019) [64] observed that seeds inoculated with *B. cereus* significantly increased the mean values of growth traits, inducing drought tolerance, possibly due to the ability of the bacterium to solubilize P, K, and Zn, thus contributing to improved plant nutrition and promoting healthier growth and development, with a consequent increase in stress tolerance [65].

In the present study, inoculation with *B. cereus* in popcorn hybrid UENF WS01 resulted in beneficial effects on several morphological plant growth traits, including LW, LB, SB, and SLA. Our results indicate significant increases in these traits (*p* < 0.01), highlighting the potential of bioinoculation to promote plant development under water stress conditions. These findings underscore the importance of microbial inoculants in enhancing plant resilience and growth performance during periods of limited water availability. Stomatal and epidermal cell density increased due to water stress, indicating a form of adaptation to water scarcity. Hussain et al. (2019) [66] and Song et al. (2020) [67], in studies on drought and heat tolerance in *Zea mays*, found that increases in stomata and epidermal cells in stressed corn plants were associated with improved water use efficiency and the ability to tolerate adverse conditions. These adaptations enable corn plants to maximize water absorption from the soil and minimize water loss through transpiration, ensuring survival under adverse environmental conditions [66].

Among the various root traits evaluated, water stress significantly increased only in section **e**, which extended from 120 to 150 cm of the specific length. Roots play a critical role in sensing water deficit signals and the absorption of water and nutrients [68]. From germination, roots primarily capture water and nutrients essential for plant development and growth [69]. The observed increase in the specific length of this root section is attributed to the production of hormones by bacteria, which stimulate root volume expansion and enable greater soil volume exploration, optimizing water absorption as an adaptive strategy to water deficit conditions [70].

In this study, bacterial inoculation increased the biomass of both the shoots and the deepest portion of the root system of popcorn plants. This enhancement may be related to increased root length and the improved formation of lateral roots and root hairs, responding to the need for water and nutrient absorption in plants exposed to water stress. The impacts of bioinoculation on morphological and physiological traits highlight the various mechanisms through which *B. cereus* positively influences plant growth and development, even under challenging environmental conditions.

Identifying morphophysiological variability in popcorn inoculated with *B. cereus* holds promise for breeding programs and biotechnological initiatives to develop future biotechnological products, such as bioinoculants. By leveraging the beneficial effects of *B. cereus* on plant morphophysiology for stress tolerance, plant breeders, physiologists, and biotechnologists are poised to contribute to the development of fortified cultivars that are resilient and can thrive in adverse conditions, thus positively impacting agricultural sustainability and food security.

## 5. Conclusions

The conclusion of this study highlights the effectiveness of inoculation with *Bacillus cereus* in promoting morphophysiological adaptations in popcorn plants (UENF WS01 genotype) under water deficit conditions. Inoculated plants showed significant improvements in various parameters, such as relative chlorophyll content, maximum fluorescence intensity, chlorophyll fluorescence, stem diameter, leaf area, root length and density, increased biomass, and improved water use efficiency, suggesting a crucial role of this bacterium in mitigating the negative effects of water stress. Furthermore, the increase in stomatal density and epidermal cells, along with the strengthening of the root system, reinforces the adaptive capacity of plants in the face of water scarcity.

These results indicate great potential to use *B. cereus* in crop improvement programs and to develop bioinoculants, contributing to enhanced plant resilience in adverse environments and promoting more sustainable agriculture. The application of these technologies can, therefore, be a valuable tool for improving food security in the context of climate change.

## Data Availability

The authors can provide experimental data for all interested researchers.

## Supplementary Materials

The following supporting information can be downloaded at https://www.mdpi.com/article/10.3390/microorganisms12112351/s1: Figure S1. Soil water potential (-MPa) as a function of days after planting (DAP) popcorn hybrid UENF WS01 under well-watered (solid blue line) and water stress (solid red line) conditions; Figure S2. Average temperature (°C) (red dotted line), relative humidity (%) (blue dashed line), and photosynthetically active radiation (µmol m^−2^ s^−1^) (green solid line) recorded by the meteorological station throughout the experiment (February to March 2023); Table S1. Summary of individual and combined analyses of variance, mean estimates, standard deviations, and coefficients of experimental variation for 19 physiological traits evaluated in the UENF WS01 popcorn hybrid, grown under full irrigation (WW) and water deficit (WS) conditions with *Bacillus cereus* inoculation; Table S2. Summary of individual and combined analyses of variance, mean estimates, standard deviations, and coefficients of experimental variation for 19 morphological traits evaluated in the UENF WS01 popcorn hybrid, grown under full irrigation (WW) and water deficit (WS) conditions with *Bacillus cereus* inoculation.
