# Supplementary material for: Bacillus cereus: An Ally Against Drought in Popcorn Cultivation
Source: Microorganisms. 2024 Nov 18;12(11):2351. doi: 10.3390/microorganisms12112351 (PMC11596106; doi:10.3390/microorganisms12112351)
Supplement: Supplementary file 1 [file microorganisms-12-02351-s001.zip › microorganisms-3273167-supplementary.pdf]

microorganisms

MDPI

Supplementary material

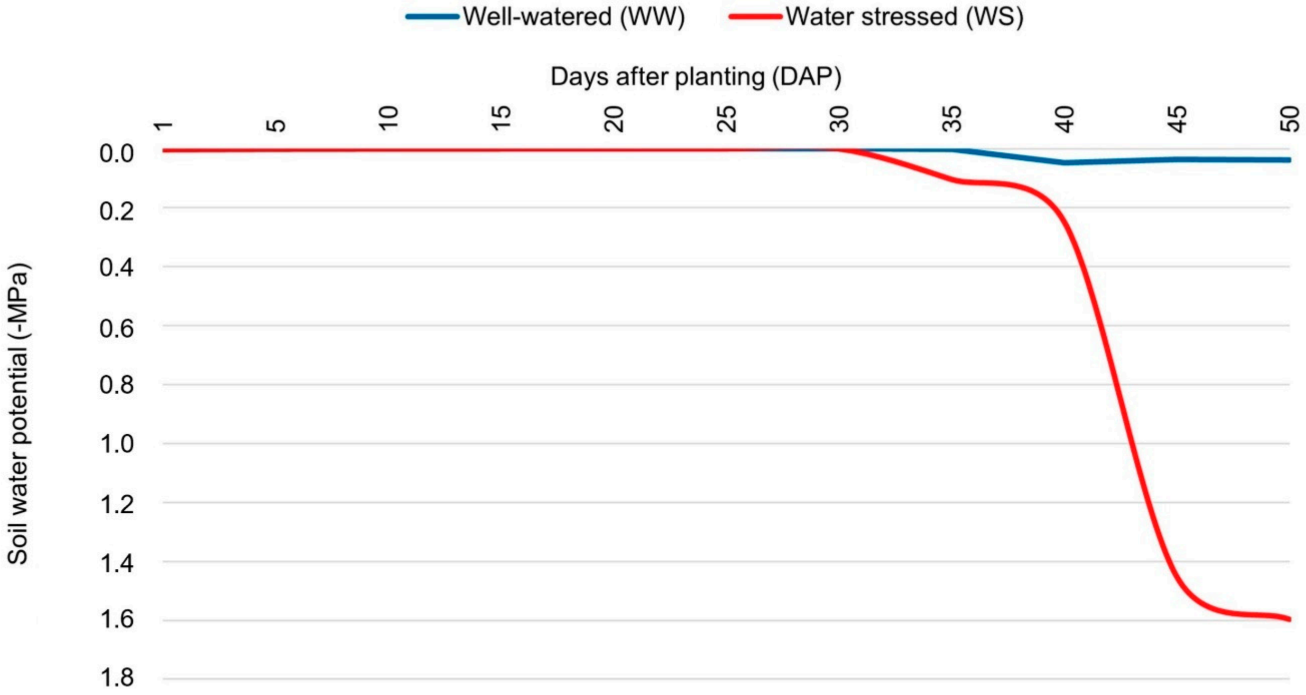

Figure S1. Soil water potential (-MPa) as a function of days after planting (DAP) popcorn hybrid UENF WS01 under well-watered (solid blue line) and water stress (solid red line) conditions.

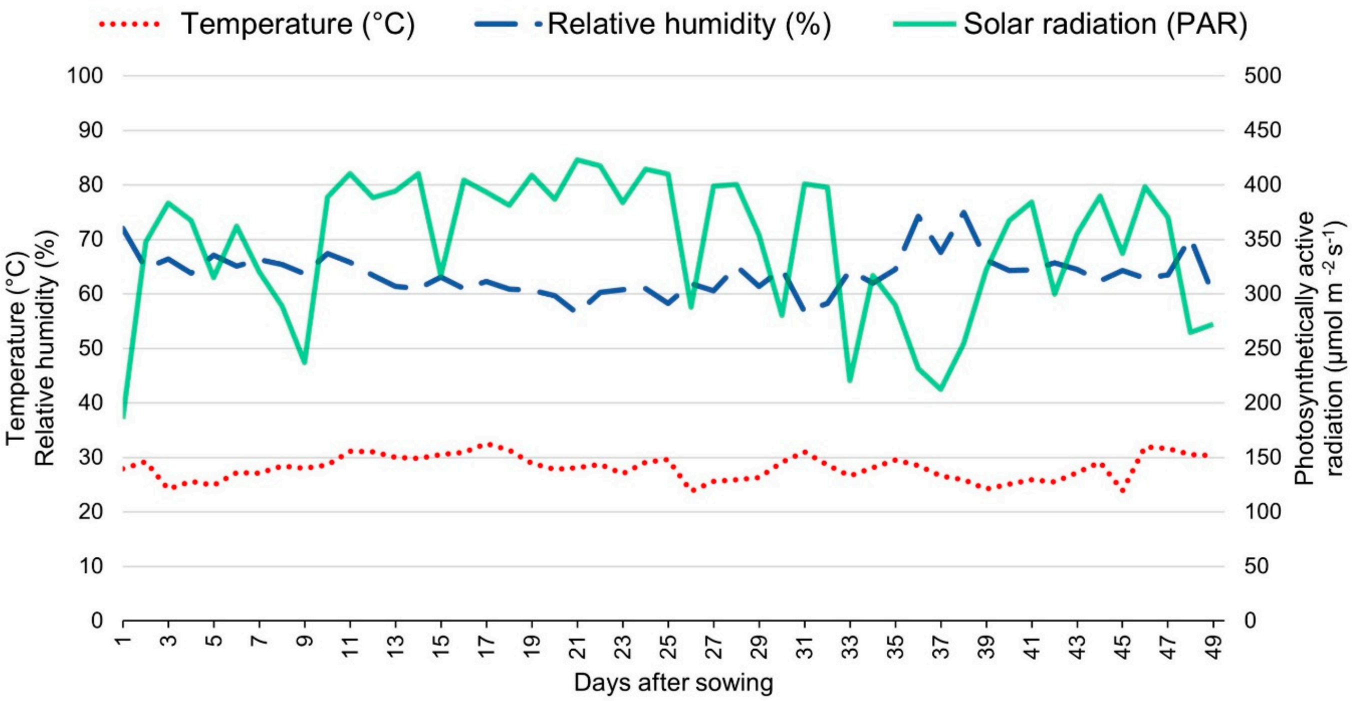

Figure S2. Average temperature (ºC) (red dotted line), relative humidity (%) (blue dashed line), and photosynthetically active radiation (μmol m^{-2} s^{-1}) (green solid line) recorded by the meteorological station throughout the experiment (February to March 2023).

Table S1. Summary of individual and combined analyses variance, mean estimates, standard deviations, and coefficients of experimental variation for 19 physiological traits evaluated in the UENF WS01 popcorn hybrid, grown under full irrigation (WW) and water deficit (WS) conditions associated with *Bacillus cereus* inoculation.

Trait	T	Water condition		CV_e (%)	Combined		
		WS	WW				
		Mean ± SD	Mean ± SD		BC	WC	BC*WC
Chl	I	19.10 ± 1.74	36.44 ± 2.39	7.67	**	**	**
	C	12.70 ± 2.43	34.04 ± 1.91	5.30			
Flav	I	0.9967 ± 0.03	0.8256 ± 0.02	2.70	**	**	**
	C	0.8578 ± 0.01	0.7444 ± 0.02	2.38			
Anth	I	0.3700 ± 0.02	0.2000 ± 0.01	7.99	*	**	*
	C	0.2922 ± 0.03	0.1756 ± 0.01	4.21			
NBI	I	20.64 ± 1.33	48.99 ± 4.77	8.26	**	**	ns
	C	14.46 ± 1.33	41.72 ± 4.86	6.33			
Fm	I	72265.33 ± 15185.70	70697.67 ± 2015.85	10.62	**	**	**
	C	57700.22 ± 8354.61	70774.33 ± 2375.96	1.98			
Fv	I	48761.22 ± 8235.60	54521.78 ± 2252.14	16.04	ns	**	ns
	C	44011.11 ± 10508.20	54412.67 ± 3647.61	5.23			
Fm/Fo	I	3.81 ± 0.54	4.59 ± 0.36	30.25	*	**	ns
	C	2.70 ± 1.17	4.33 ± 0.36	6.64			
Fv/Fo	I	3.11 ± 0.94	3.63 ± 0.33	40.59	**	**	*
	C	1.74 ± 1.14	3.33 ± 0.36	8.75			
Fv/Fm	I	0.76 ± 0.07	0.78 ± 0.01	14.87	*	*	ns
	C	0.65 ± 0.13	0.77 ± 0.02	2.04			
A	I	20.46 ± 2.47	25.87 ± 1.79	5.34	**	**	ns
	C	16.98 ± 1.77	23.18 ± 4.17	13.31			
Gs	I	0.17 ± 0.01	0.22 ± 0.02	9.59	**	**	ns
	C	0.11 ± 0.02	0.18 ± 0.05	19.98			
Ci	I	139.25 15.06	172.70 18.44	14.28	*	**	ns
	C	114.51 21.79	154.84 32.36	17.52			
E	I	2.01 ± 0.61	2.81 ± 0.68	9.62	ns	**	ns
	C	1.72 ± 0.48	2.59 ± 0.76	20.44			
NPQT	I	3.78 ± 2.04	1.94 ± 0.63	65.81	ns	*	ns
	C	3.46 ± 2.86	1.76 ± 0.77	38.34			
PHI2	I	0.47 ± 0.08	0.55 ± 0.05	27.58	ns	**	ns
	C	0.42 ± 0.17	0.55 ± 0.07	13.73			
RLWC	I	86.47 ± 8.63	90.87 ± 1.76	10.22	**	ns	ns
	C	74.13 ± 4.81	78.51 ± 12.24	9.96			
WUEint	I	132.42 ± 12.65	116.81 ± 17.47	7.60	*	**	ns
	C	156.86 ± 13.34	123.84 ± 25.03	18.27			
WUEinst	I	9.66 ± 1.51	9.84 ± 0.86	11.20	*	ns	ns
	C	11.03 ± 1.86	10.13 ± 1.45	8.96			

WUEagr	I	5.03 ± 0.46	5.96 ± 0.69	18.81	ns	**	ns
	C	4.86 ± 0.92	6.05 ± 0.73	9.92			

**, *, ns, significant at 1%, 5%, and not significant, respectively, by the F test. WS: water stress condition; WW: well-watered condition; SD: standard deviation; CVe: coefficient of experimental variation; BC: bacterium; WC: water condition; BC*WC: bacterium*water condition interaction.

Relative chlorophyll content (Chl), leaf anthocyanin content (Anth), flavonoid content (Flv), nitrogen balance index (NBI), maximum fluorescence intensity (Fm), variable fluorescence (Fv), basal quantum production of non-photochemical processes in PSII (Fm/Fo), potential quantum efficiency of PSII (Fv/Fo), potential quantum yield (Fv/Fm), net photosynthetic rate (A), stomatal conductance (GS), intercellular CO_2 concentration (Ci), transpiration rate (E), non-photochemical quenching (NPQT), quantum yield of PSII (PHI2), relative leaf water content (RLWC), intrinsic water use efficiency (WUEInt), instantaneous water use efficiency (WUEinst), and agronomic water use efficiency (WUEagro).

Table S2. Summary of individual and combined analyses of variance, mean estimates, standard deviations, and coefficients of experimental variation for 19 morphological traits evaluated in the UENF WS01 popcorn hybrid, grown under full irrigation (WW) and water deficit (WS) conditions associated with *Bacillus cereus* inoculation.

Trait	T	Water condition		CV_e (%)	Combined		
		WS	WW		BC	WC	BC*WC
		Mean ± SD	Mean ± SD				
PH	I	57.27 ± 4.47	92.72 ± 4.23	5.42	ns	**	ns
	C	55.20 ± 5.21	92.58 ± 7.59	5.50			
SD	I	13.79 ± 0.82	15.09 ± 0.50	6.23	ns	**	ns
	C	13.58 ± 0.82	14.94 ± 0.58	3.25			
LL	I	70.78 ± 10.07	80.06 ± 3.59	7.94	*	*	ns
	C	76.70 ± 6.11	82.70 ± 4.16	3.87			
LW	I	6.70 ± 0.11	7.04 ± 0.17	1.78	**	**	ns
	C	6.42 ± 0.19	6.75 ± 0.21	3.07			
LB	I	9.69 ± 1.10	12.53 ± 0.82	11.75	**	**	ns
	C	8.55 ± 0.64	11.53 ± 0.85	6.42			
SB	I	7.96 ± 0.61	14.83 ± 1.30	4.89	**	**	ns
	C	6.62 ± 0.59	13.54 ± 0.63	7.27			
SLA	I	228.37 ± 10.67	225.86 ± 17.23	4.88	**	ns	ns
	C	244.22 ± 12.17	247.28 ± 13.65	5.93			
SDAB	I	150.90 ± 22.16	106.44 ± 21.15	10.34	*	**	ns
	C	135.40 ± 22.52	99.54 ± 16.61	14.07			
ECDAB	I	750.27 ± 237.25	527.87 ± 76.13	18.34	ns	**	ns
	C	743.21 ± 140.17	562.96 ± 65.31	11.37			
SDAD	I	113.74 ± 19.56	94.89 ± 18.93	8.95	*	ns	ns
	C	99.15 ± 15.56	81.02 ± 10.81	19.95			
ECDAD	I	609.35 ± 100.42	502.82 ± 67.28	18.34	ns	*	ns
	C	567.90 ± 95.84	540.56 ± 74.10	15.24			
SIAB	I	22.54 ± 5.78	20.65 ± 1.95	20.69	**	**	ns
	C	18.79 ± 3.97	18.65 ± 1.35	8.81			
SIAD	I	20.43 ± 2.08	21.32 ± 3.95	11.57	**	ns	ns
	C	18.15 ± 4.05	15.09 ± 2.30	19.61			
MNRa	I	68.20 ± 5.45	62.20 ± 5.81	7.85	*	ns	ns

	C	59.60 ± 3.21	59.40 ± 5.86	9.57			
MNRb	I	31.00 ± 6.12	24.20 ± 3.11	22.37	ns	**	ns
	C	27.40 ± 5.55	21.20 ± 2.68	15.88			
MNRc	I	20.80 ± 1.48	20.40 ± 1.67	11.02	ns	**	ns
	C	20.20 ± 2.17	20.20 ± 2.39	12.66			
MNRd	I	18.00 ± 1.22	16.80 ± 1.92	19.98	ns	*	ns
	C	17.60 ± 4.16	13.60 ± 0.89	11.58			
SRLe	I	3822.67 ± 1829.94	1779.97 ± 204.94	30.38	ns	*	ns
	C	3274.19 ± 1328.38	1482.19 ± 219.57	11.31			
RWDc	I	1.74 ± 0.31	5.38 ± 2.19	12.32	**	**	*
	C	1.39 ± 0.38	1.55 ± 0.72	48.00			

**, *, ns, significant at 1%, 5%, and non-significant, respectively, by the F test. WS: water stress condition; WW: well-watered condition; SD: standard deviation; CVe: coefficient of experimental variation; BC: bacterium; WC: water condition; BC*WC: bacterium*water condition interaction.

Mean plant height (PH), mean stem diameter (SD), mean leaf length (LL), mean leaf width (LW), mean leaf biomass (LB), mean stem biomass (SB), specific leaf area (SLA), abaxial stomata density (SDAB), abaxial epidermal cell density (ECDAB), adaxial stomata density (SDAD), adaxial epidermal cell density (ACDAD), abaxial stomatal index (SIAB), adaxial stomatal index (SIAD), mean number of roots (MNR, section **a**, **b**, **c**, and **d**), specific root length (SRLe), and root weight density (RWDc).
